# Immunoadsorption versus double-dose methylprednisolone in refractory multiple sclerosis relapses

**DOI:** 10.1186/s12974-022-02583-y

**Published:** 2022-09-07

**Authors:** Steffen Pfeuffer, Leoni Rolfes, Timo Wirth, Falk Steffen, Marc Pawlitzki, Andreas Schulte-Mecklenbeck, Catharina C. Gross, Marcus Brand, Stefan Bittner, Tobias Ruck, Luisa Klotz, Heinz Wiendl, Sven G. Meuth

**Affiliations:** 1 Department of Neurology and Institute of Translational Neurology, University Hospital Muenster, Albert-Schweitzer-Campus 1, 48149 Muenster, Germany; 2 Department of Neurology, University Hospital Mainz, Mainz, Germany; 3 Department of Neurology, University Hospital Duesseldorf, Duesseldorf, Germany; 4 Medical Department D - Nephrology, University Hospital Muenster, Muenster, Germany

**Keywords:** Multiple sclerosis, Relapse, Immunoadsorption, Intravenous methylprednisolone, Steroids

## Abstract

**Objective:**

Intravenous methylprednisolone is the standard treatment for a multiple sclerosis relapse; however, this fails to improve symptoms in up to one quarter of patients. Immunoadsorption is an accepted treatment for refractory relapses, but prospective comparator-controlled studies are missing.

**Methods:**

In this observational study, patients with steroid-refractory acute multiple sclerosis relapses receiving either six courses of tryptophan-immunoadsorption or double-dose methylprednisolone therapy were analysed. Outcomes were evaluated at discharge and three months later. Immune profiling of blood lymphocytes and proteomic analysis were performed by multi-parameter flow cytometry and Olink analysis, respectively (NCT04450030).

**Results:**

42 patients were enrolled (methylprednisolone: 26 patients; immunoadsorption: 16 patients). For determination of the primary outcome, treatment response was stratified according to relative function system score changes (“full/best” vs. “average” vs. “worse/none”). Upon discharge, the adjusted odds ratio for any treatment response (“full/best” + ”average” vs. “worse/none”) was 10.697 favouring immunoadsorption (*p* = 0.005 compared to methylprednisolone). At follow-up, the adjusted odds ratio for the best treatment response (“full/best” vs. “average” + ”worse/none”) was 103.236 favouring IA patients (*p* = 0.001 compared to methylprednisolone). Similar results were observed regarding evoked potentials and quality of life outcomes, as well as serum neurofilament light-chain levels. Flow cytometry revealed a profound reduction of B cell subsets following immunoadsorption, which was closely correlated to clinical outcomes, whereas methylprednisolone had a minimal effect on B cell populations. Immunoadsorption treatment skewed the blood cytokine network, reduced levels of B cell-related cytokines and reduced immunoglobulin levels as well as levels of certain coagulation factors.

**Interpretation:**

Immunoadsorption demonstrated favourable outcomes compared to double-dose methylprednisolone. Outcome differences were significant at discharge and follow-up. Further analyses identified modulation of B cell function as a potential mechanism of action for immunoadsorption, as reduction of B cell subsets correlated with clinical improvement.

**Supplementary Information:**

The online version contains supplementary material available at 10.1186/s12974-022-02583-y.

## Introduction

Despite significant advances in long-term immunomodulatory therapies for multiple sclerosis (MS), treatment for acute MS relapses has remained largely unaltered for the past 20 years [1]. International guidelines recommend the administration of high-dose intravenous methylprednisolone (MPS), up to 1000 mg daily for three to five consecutive days, for the alleviation of symptoms [2, 3]. However, approximately 25% of patients respond insufficiently to the first cycle of MPS [4] and current guidelines recommend a second, double-dose cycle of up to 2000 mg daily for three to five consecutive days [5]. Therapeutic apheresis for the clearance of soluble plasma components has entered clinical routine as an alternative, including therapeutic plasma exchange as well as immunoadsorption (IA) [6, 7]. Although not yet evaluated in randomized clinical trials, IA has been proven to be effective in two prospective [8, 9] and several retrospective studies [10–13], reporting response rates between 50 and 86% among patients suffering from isolated optic neuritis, clinically isolated syndrome, or relapsing MS (RMS) who had previously responded insufficiently to MPS[8, 10–13]. Consequently, several guidelines recommend IA as an adjunctive [14] treatment to increasing the chance of recovery from steroid-refractory relapses [2].

Apheresis treatment is, however, invasive—often requiring the insertion of a central venous catheter—and more expensive compared to MPS. In addition, there is no comparison in the literature between the efficacy of any kind of apheresis treatment and a second cycle of double-dose MPS.

Additionally, substantial differences exist between both treatment regimens in terms of mechanism of action. MPS treatment is thought to target T cells almost exclusively by induction of apoptosis and exert only a minor effect on soluble factors [15]. IA, however is supposed to largely modulate soluble but not cellular factors [6]. However, little is known how immunoadsorption alleviates relapse symptoms in detail and thus, predictors for treatment response are unknown yet desired given the abovementioned risks for treatment.

Therefore, we conducted a prospective clinical study comparing the clinical outcomes of a second course of MPS versus six courses of tryptophane column-based IA in patients refractory to the initial cycle of MPS for the treatment of an acute MS relapse. Furthermore, we performed extensive analyses of cellular and soluble factors in peripheral blood to further elucidate the impact of either treatment on the immune system.

## Methods

### Patients

INCIDENT-MS (ImmuNoadsorption versus high-dose intravenous CorticosteroIDs in RElapsing Multiple Sclerosis—AssessmenT of MechaniSm of Action) was designed as a larger prospective observational study to assess the safety and efficacy of immunoadsorption versus methyl prednisolone for refractory MS relapses and to evaluate the mechanism of action for each treatment. Recruitment included all patients admitted to the Department of Neurology, University Hospital Muenster, Germany.

Patients that underwent a first course of intravenous methylprednisolone (MPS) (1000 mg per day for five consecutive days; “initiation treatment”) yet experienced persistent deficits were identified and were offered to receive either another course of MPS (2000 mg per day for five consecutive days) or tryptophan immunoadsorption (six courses every other day; “escalation treatment”). Treatment regimen was determined by shared-decision making following thorough information of patients by consultants not connected to the study. Patients were enrolled from August 2018—August 2020. In- and exclusion criteria are listed in Table 1.

**Table 1:** In- and exclusion criteria of the INCIDENT-MS study

**Inclusion criteria**
• Signed informed consent form
• Established diagnosis of relapsing MS according to the 2017 revised McDonald-criteria
• Incomplete remission of symptoms after administration of 1000 mg intravenous (methyl-) prednisolone as measured by the EDSS value: •EDSS value baseline + 1, if pre-treatment EDSS value is ≤ 3.5; EDSS value baseline + 0.5, if pre-treatment EDSS value is > 3.5
• Absence of clinically apparent fever or concomitant infection. Asymptomatic urinary tract infection is not considered as significant infection unless it leads to an at least two-fold increase of C-reactive protein levels above ULN (upper level of normal)
**Exclusion criteria**
• Patients with a documented EDSS > 6.5 prior to recent relapse. Patients that are suspicious to having entered a secondary-progressive course of the disease at the time point of screening
• Patients that previously received either escalation treatment for refractory MS relapses
• Female patients known to be pregnant or unwilling to perform a pregnancy test
• Patients that receive immunosuppressive treatment for diseases other than RRMS or that receive long-term corticosteroid treatment
• Patients that received less than 3 g or more than 5 g (methyl-)prednisolone prior to initial admission or that received (methyl-)prednisolone for more than 8 days
• Patients with verified infection by human-immunodeficiency-virus or hepatitis-c-virus
• Patients with medical, psychiatric, cognitive, or other conditions that, in the investigator’s opinion, compromise the patient's ability to understand the patient information, to give informed consent, or to complete the study
• Patients with significant psychiatric comorbidities with the necessity of specific treatment during administration of intravenous steroids at the investigators discretion
• Patients on regular medication with inhibitors of angiotensin-converting-enzyme (ACE) inhibitors
• Patients with major impairment of the blood coagulation system with increased risk during establishment of central venous catheters as follows: •therapy with anticoagulants for any purpose other than prevention of deep vein thrombosis •elevation of INR above 1.5, elevation of PTT above 50 s •thrombocytopenia below 50.000/μL •intake of dual antiplatelet therapy

*MS* multiple sclerosis, *NMOSD* neuromyelitis optica-spectrum disorder, *CRION* chronic-relapsing inflammatory optic neuropathy, *EDSS* expanded disability status scale, *ON* optic neuritis, *ULN* upper limit of normal, *ACE* angiotensin converting enzyme, *INR* international normalized ratio, *PTT* partial thromboplastin time

Initially, the study was designed to incorporate a larger cohort of 204 patients in order to allow confirmation of various secondary endpoints as well as immunologic analyses but health insurances temporarily halted reimbursement of IA treatment for RMS patients. Thus, we here present results from the core study population which were sufficient to evaluate the primary outcome at common significance parameters (α level: 0.05, power: 80%).

Within the core population, a minimal sample size of 15 patients per group was calculation upon results from a previous retrospective analysis of patients undergoing treatment for refractory MS relapses in our department [7]. However, the decision was made to include all suitable patients during the core study period to avoid selection bias, even if 15 patients per group were exceeded.

### Treatment

MPS (2000 mg per day for five consecutive days) was administered according to clinical guidelines and was accompanied by prophylaxis against gastric ulcers, venous thrombosis and osteoporosis.

IA was performed using jugular central venous catheters. Plasma separation was performed using the Octo Nova extracorporeal circuit technology (SV 4.30.6, Front 4.30.6) and the polyethylene plasma separator OP-05W (Asahi Kasei Kuraray, Tokyo, Japan). Plasma filtrate passes through a tryptophan column (Immunosorba TR-350, Diamed, Germany). For all treatments unfractionated heparin was used for anticoagulation. Six sessions with a treated plasma volume of 2.5 L were performed within 6–8 days.

### Outcome measurements

Patients were examined by two trained neurologists at baseline (prior to initiation of escalation treatment), including the assessment of expanded disability status scale (EDSS) score, multiple sclerosis functional composite (MSFC) testing, and the SF36 questionnaire to assess health-related quality-of-life (QoL). Relapses were determined to be either “visual”, “motor” or “sensory”, depending on the clinical presentation. Within the first two days, patients underwent electrophysiology testing, including evoked potentials (full-field visual-evoked potentials and somatosensory-evoked potentials from tibial nerves). These tests were repeated at discharge as well as at three-month follow-up.

Physicians involved in testing were given no information regarding treatment and CVC were removed prior testing in IA patients. All tests were conducted in a standardized environment at our scientific outpatient clinic for further reduction of bias. Patients were screened for adverse events daily. Patients with incomplete remission following escalation treatment were offered to receive the respective treatment as rescue therapy. Patients undergoing this procedure were again examined as described above at the beginning and end of rescue therapy.

For determination of the treatment response, we performed analysis of EDSS function score changes according to the system proposed by Conway and colleagues [17]. Briefly, treatment response was stratified according to relative function system score changes (“full/best” vs. “average” vs. “worse/none”) and proportions of patients within each response group were compared. We defined the proportion of patients with “full/best” + ”average” recovery vs. the proportion of patients with “worse/none” response per treatment group assessed upon discharge from escalation treatment as primary endpoint. Secondary endpoints comprised the treatment response at follow-up, EDSS scores at discharge and follow-up, MSFC scores, SF-36 scores, and evoked potentials. MSFC and SF-36 results were interpreted using the reference manuals. Evoked potential outcomes were categorized using a six-step ordinal system as described by Jung et al. [16]. Tertiary analyses comprised evaluation of cellular and soluble factors from peripheral blood (see below).

### Multiparameter flow cytometry

Peripheral blood mononuclear cell (PBMC) samples analyzed by flow cytometry were generated by density gradient centrifugation using Lymphoprep (Stemcell technologies) and subsequent cryo-preservation in serum-free medium (CTL-Cryo ABC Media Kit, Immunospot) in the vapor phase of a liquid nitrogen tank.

For flow cytometry, PBMC were thawed by placing in a 37 °C water bath for 8 min. The cell suspension was transferred to a 50 ml conical tube and 9 ml pre-warmed RPMI-medium (RPMI (Sigma Aldrich), 10% FCS Gold Plus (BioSell), 1% Glutamax (Gibco), 1% Na-Pyruvate (invitrogen)) was added prior to centrifugation at 300*g* for 10 min. Supernatant was discarded and the cell pellet was resuspended in RPMI-medium. PBMC were counted and viability was assessed using a Countess II automated cell counter (Invitrogen). Subsequently, PBMC were subjected to immune phenotyping by flow cytometry. Therefore, PBMC were directly stained with fluorochrome-conjugated antibodies (for complete list see Additional file 1: Table S1) In addition, intra-cellular/-nuclear epitopes were investigated by incubation of PBMC with Perm/Fix buffer (BD Biosciences) for 20 min at room temperature and subsequent staining for 30 min at 4 °C in Perm buffer (BD Biosciences). Samples were acquired on a Cytoflex 13-color flow cytometer (Beckman Coulter) under daily quality control by CytoFlex Daily QC Fluorospheres (Beckman Coulter). Resulting data was analyzed using Kaluza 2.1 (Beckman Coulter) by manual gating on PBMC subsets. Absolute cell counts were calculated from differential blood counts which were acquired during clinical routine upon sampling of study blood.

### Serum analysis

Serum samples were collected following standard procedure. After 30–45 min (min) at room temperature, separation of serum was achieved by differential centrifugation at 2000*g* for 10 min at room temperature. Samples were aliquoted in polypropylene tubes and stored at − 80 °C until further analysis. sNfL was measured by single molecule array with a SiMoA HD-1 (Quanterix) using the NF-Light Advantage Kit (Quanterix) according to the manufacturer’s instructions. Samples were measured in duplicate. Blinded sNfL measurements were performed, without information about clinical data. For cytokine analysis, serum samples were sent to Olink (Uppsala, Sweden) using the “Olink target 48-cytokine” assay containing 45 selected cytokines (see Additional file 1: Table S2).

### Statistical analysis

Epidemiological data at baseline were analysed using descriptive statistics and comparisons among groups were made using the Mann–Whitney U test or the Kruskal–Wallis test for continuous variables and Fisher’s exact test for categorical variables. To assess recovery, function system scores at baseline and discharge were categorized as “best”, “average”, or “worse”, according to previous work published by Conway and colleagues [17]. For adjustment, logistic regression models were established using “best/average vs. worse recovery” (discharge) or “best vs. average/worse recovery” (follow-up) as dependent variables and “sex (male/female)”, “baseline-EDSS”, “affected function system (visual/motor/sensory)”, and “first demyelinating event (yes/no)” as covariates in an enter method (with p-values derived from a likelihood-ratio test). Differences of the MSFC at discharge or follow-up compared to baseline were analysed using linear regression models using these covariates.

Experimental data were analysed using the Mann–Whitney *U* test or Kruskal–Wallis test including Dunn’s post-test. For comparison of longitudinal data sets, Friedman’s test was used. Volcano plots were generated by plotting log2 values of the relative difference between the medians (continuous) or means (categorical parameters) against the p-values, calculated using the Mann–Whitney *U* test. Outside from pre-defined clinical endpoints (proportion of patients with a response to treatment), data were considered exploratory. Bonferroni-correction was applied to immunologic analysis where appropriate and is shown in the respective figures. A *p*-value below 0.05 was considered significant.

### Ethical approval and study registration

Ethical approval was given by local authorities (Medical Council Westphalia-Lippe; 2018-261-f-S) and the study was listed in the National Institute of Health’s registry [clinicaltrials.gov; NCT04450030].

### Consent for publication

Not applicable.

### Data availability statement

Anonymized data will be shared with any qualified investigator upon reasonable request.

## Results

### Patients

**Fig.1:**
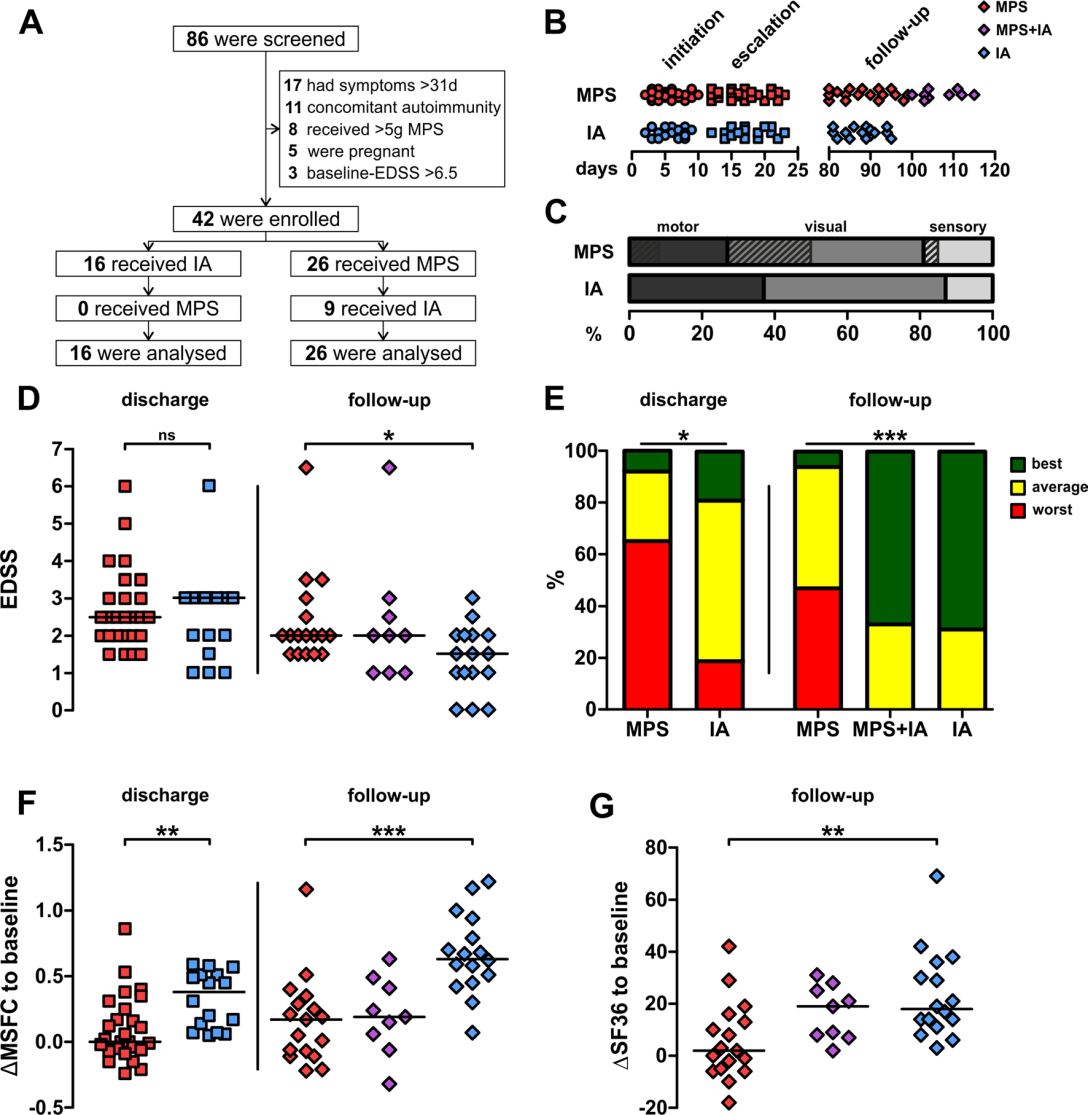
Clinical outcomes of the INCIDENT-MS study **A** CONSORT plot indicating patient groups. **B** Dot plot indicating duration of initiation therapy (circles), escalation therapy (boxes) and follow-up (diamonds) from relapse onset. Colours indicate different treatment groups and can be referred to throughout. **C** Bar graphs indicating the proportion of different relapse categories among treatment groups. Shaded boxes indicate patients who received immunoadsorption as second-escalation therapy. **D** EDSS scores among treatment groups at discharge (boxes) and follow-up (diamonds). **E** Treatment response stratified according to the matrix proposed by Conway et al. at discharge and follow-up. **F** Difference of the MSFC scores at discharge and follow-up compared to baseline. **G** Difference of SF-36 healthcare-related quality of life scores at follow-up compared to baseline. *: *p* < 0.05; **: *p* < 0.01; ***: *p* < 0.001; significance levels were determined using Mann–Whitney rank sum test (**D**; **F**; **G**) or Fisher’s exact test (**E**). *IA* immunoadsorption, *MPS* methylprednisolone, *EDSS* expanded disability status scale; *MSFC* multiple sclerosis functional composite, *SF36* short-form 36 questionnaire

Between August 2018 and June 2020, 86 patients with RMS were screened and 42 patients were enrolled (Fig. 1A). 26 (72%) patients received a second course of MPS, whereas 16 (38%) patients were treated with IA. Due to incomplete clinical recovery, 9/26 (35%) patients were subjected to IA as second escalation therapy (“MPS + IA”). None of the IA patients required a second course of MPS (Fig. 1A).

Baseline epidemiological characteristics were balanced among treatment groups (Table 2). All patients fulfilled the 2017 revised McDonald criteria [18] for the diagnosis of RMS at baseline. Overall, patients were young (median age: 35 years) and early in their disease course, reflected by the absence of previous demyelinating events in 57% of patients. 71% of patients were treatment-naïve, 7% received treatment approved for mild to moderate disease (glatiramer acetate: two patients; beta-interferon: one patient) and 22% received treatment for active forms of RMS (natalizumab: six patients; fingolimod: three patients). None of the patients received cell-depleting therapies. Administration of disease-modifying treatment was equally distributed among groups (*p* = 0.869; Table 2).

**Table 2:** Baseline characteristics of the enrolled patients

	MPS (*n* = 26)	IA (*n* = 16)	*p*
Age, yrs, median (IQR)	38 (27–47)	32 (23–45)	0.265*
Female patients, n (%)	19 (73)	10 (63)	0.510^#^
EDSS at baseline (IQR)	2.5 (2–3.5)	3 (2–4)	0.112*
Patients with first demyelinating event, *n* (%)	15 (65)	9 (56)	0.745^#^
Patients with DMT, *n* (%)			
None	19 (73)	11 (69)	
Basic	2 (8)	1 (6)	
Escalation	5 (19)	4 (25)	0.869^#^
Duration since relapse onset, days, median (IQR)			
To initiation therapy	5(4–7)	6 (4–8)	0.592*
To escalation therapy	17 (15–20)	17 (15–21)	0.507*
Duration from discharge to follow-up, days, median (IQR)	94 (85–103)	89 (84–94)	0.102*
Relapse category, *n* (%)			
Visual	14 (54)	8 (50)	
Motor	7 (27)	6 (38)	
Sensory	5 (19)	2 (13)	0.758^#^

*MPS* methylprednisolone, *IA* immunoadsorption. *IQR* interquartile range, *EDSS* expanded disability status scale, *DMT* disease-modifying treatment. Continuous data were evaluated using the Mann–Whitney rank sum test (*). Categorical data were tested using Fisher’s exact test (^#^)

The latencies between onset of relapse and the start of initial MPS therapy, as well as between onset of relapse and escalation therapy with either high-dose MPS or IA, were similar in both groups. Of note, time from symptom onset to follow-up was longer in MPS + IA patients compared to MPS patients (median, days (interquartile range): MPS: 88 (83–94); MPS + IA: 104 (103–111), *p* < 0.01; Fig. 1B).

The most common relapse presentation was optic neuritis (22 patients, 52%). The frequency of a given functional system being affected was evenly distributed between both groups (*p* = 0.758; Table 2 and Fig. 1C).

All patients fully completed their escalation treatment regime and provided full clinical and laboratory datasets.

### Effectiveness outcomes

EDSS scores did not differ significantly between groups upon discharge (*p* = 0.772) but re-evaluation at follow-up yielded reduced scores among IA patients (*p* = 0.039, Fig. 1D). Following stratification of patients into treatment response groups as described above, we found a lower proportion of non-responders among IA-treated patients at discharge (*p* = 0.028), with differences persisting at follow-up. Moreover, IA as either first or second escalation treatment resulted in the complete absence of non-responders at follow-up (Fig. 1E).

Multivariate regression analysis including ‘sex’, ‘baseline-EDSS’ and ‘history of previous demyelinating events’ as covariates were generated. The adjusted odds ratio for “any treatment response at discharge” (“full/best” + ”average” vs. “worse/none”; primary outcome parameter) was 10.697 (95% CI: 2.012–56.866; *p* = 0.005) favouring IA.

At follow-up, we were first interested in the proportion of patients who made “full/best” recovery versus those, who retained disability from the current relapse (“full/best” vs. “average” + ”worse/none”). Adjusted odds ratio was 103.236 and favoured patients who underwent IA as first escalation (95% CI: 6.241–1707.627*; p* < 0.001 compared to MPS) and was 50.646 favouring patients who underwent IA as rescue treatment (95% CI: 3.115–823.150; *p* = 0.006 compared to MPS). None of the covariates were selected in either model. Linear multivariate regression evaluating MSFC scores again favoured IA at discharge (*p* = 0.040) and follow-up (*p* < 0.001) whereas MPS + IA was not significantly superior to MPS (*p* = 0.191), again without selection of covariates.

We further investigated MSFC scores in all patients and found that patients following IA had favourable outcomes at discharge (*p* = 0.002) and follow-up (*p* < 0.001). Notably, we were able to detect a substantial improvement in MSFC scores for patients having undergone IA as the first escalation treatment compared to patients having received two courses of MPS. Contrasting the observations using function score analysis, IA was not as effective as a second escalation compared to its use as a first escalation treatment, especially regarding the cognitive and upper limb function, assessed by MSFC (Fig. 1F).

SF36 questionnaire showed that patients having received IA or MPS + IA had higher scores suggestive of increased QoL at follow-up compared to patients from the MPS group (*p* = 0.005), yet no significant difference was observed between IA and MPS + IA (*p* = 0.490*;* Fig. 1G).

**Fig. 2:**
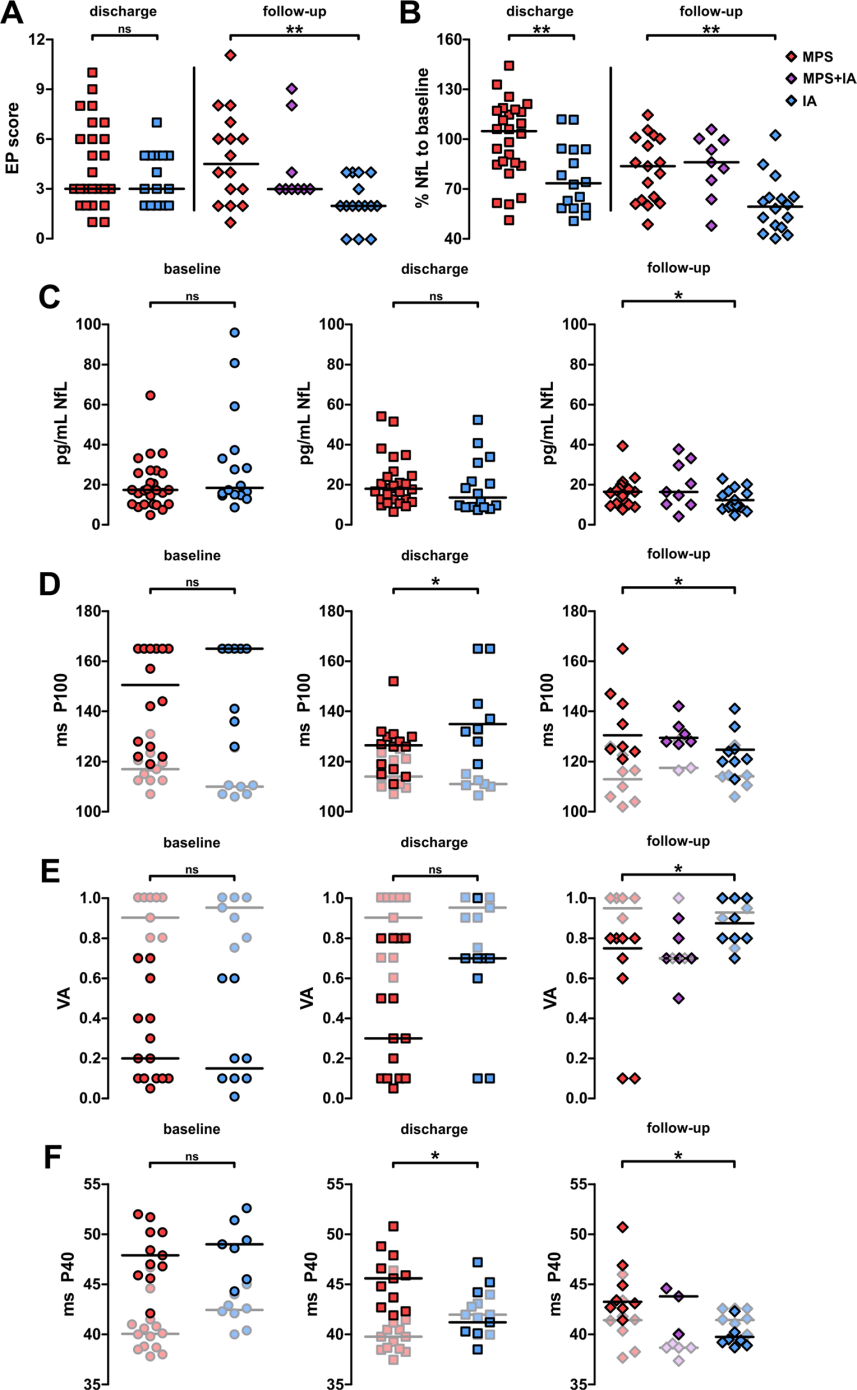
Determination of NfL serum levels, visual acuity and evoked potentials. **A** Difference of evoked potential scores at discharge (boxes; applies throughout) and follow-up (diamonds; applies throughout) compared to baseline. **B** Relative serum neurofilament light-chain levels at discharge and follow-up compared to baseline. **C:** Absolute levels of serum neurofilament light-chain levels at baseline (circles; applies throughout), discharge and follow-up. **D** Absolute values for visual-evoked potential P100 latencies among groups (affected eyes in ON patients). 40% grey symbols indicate patients with relapses other than optic neuritis (average of both eyes; applies to **E** as well). Conduction block was assumed at P100 > 165 ms. **E** Visual acuity of patients during the study (affected eyes in ON patients). **F** Absolute values for somatosensory-evoked tibial P40 latencies (body length was equally distributed among groups; *p* = 0.285). 40% grey symbols indicate patients with optic neuritis. Lines indicate median throughout. *: *p* < 0.05; ns: *p* > 0.05; significance levels were determined using Mann–Whitney rank sum test (baseline, discharge) or Kruskal–Wallis test (follow-up). *IA* immunoadsorption, *MPS* methylprednisolone, *EP* evoked potential; *NfL* neurofilament light-chain, *P100* visual-evoked potential P100 latency, *VA* visual acuity, *P40* somatosensory-evoked tibial P40 latency

In addition, patients underwent electrophysiological assessment including SEP and VEP. Although no differences were observed at discharge, the IA group performed better at follow-up (*p* = 0.009 for IA vs. MPS; *p* = 0.776 for IA vs. MPS + IA; Fig. 2A).

Serum neurofilament light-chain (NfL) levels were substantially decreased compared to baseline (median: 73%, *p* < 0.001 for longitudinal comparison) in IA patients, whereas they were rather unaffected by MPS treatment (median: 105%; *p* = 0.081 for longitudinal comparison; *p* = 0.004 for comparison of MPS and IA), although it is unknown whether NfL is retained by IA columns. However, stronger reduction was persistent in IA patients (59% of baseline) at follow-up compared to MPS (84% of baseline) or MPS + IA patients (86% of baseline; *p* = 0.002 for comparison of IA, MPS and MPS + IA); Fig. 2B). Unlike relative and longitudinal comparison, absolute NfL levels did not allow cross-sectional discrimination of patients at discharge but patients following IA presented with lower NfL levels at follow-up compared to MPS and MPS&IA patients (Fig. 2C).

Regarding evoked potentials, we found that 11 patients with optic neuritis exhibited conduction block in the affected eye. This was rapidly alleviated by treatment yet despite temporary resolution of conduction block following IVMPS, one patient experienced persistent loss of VEP at follow-up. Notably, we found only four patients with VEP alterations suggestive of previous ON among patients (Fig. 2D). Similar observations were made regarding visual acuity (Fig. 2E). Generally, correlation between VEP and acuity was high throughout the study (*p* = 0.011 for linear regression at discharge). Notably, comparison of ON and non-ON patients at follow-up showed that MPS patients had lower visual acuity and prolonged VEP latencies whereas this was not visible in IA patients. Somatosensory-evoked potentials showed relevant differences between groups already at discharge with rapid restoration of conduction in IA patients (Fig. 2F).

### Safety outcomes

26 (100%) MPS patients and 14 IA (88%) patients in the IA group experienced at least one adverse event. MPS treatment was frequently associated with hyperglycaemia (16 patients, 92%), sleep disorder (17 patients, 65%), tachycardia (13 patients, 50%), hypokalaemia (11 patients, 42%), and hypertension (five patients, 19%; Table 3).

**Table 3:** Adverse events observed throughout the study

IA	CTCAE			
	I	II	III	IV
Hypocalcaemia	3	3		
Tachycardia	2			
Hypertension	3			
Hypotension	6	2		
Hyperglycaemia	1			
Nausea	2			
CVC dislocation		2		
CVC infection			1	
necessity femoral CVC		2		

MPS	CTCAE			
	I	II	III	IV
Hyperglycaemia	8	16		
Sleep disorder	6	11		
Tachycardia	12	1		
Hypokalaemia	6	5		
Hypertension	3	2		
Oedema	1			
Liver injury			1	
Anxiety and depression	1	3		
Psychosis			1	2
Septic coxitis				1

Numbers indicate the respective events stratified according CTCAE severity scale. *IA* immunoadsorption, *CVC* central venous catheter, *MPS* methylprednisolone

Temporary insulin treatment and oral substitution of potassium was necessary in 16 patients. Four patients (15%) developed clinically significant anxiety and affective dysfunction during high-dose MPS escalation treatment, requiring the temporary use of lorazepam in two patients. One case of MPS-induced liver injury was observed, which resolved within two weeks with conservative management. Osteomyelitis of the left hip with subsequent septicaemia developed in an otherwise healthy 26-year-old following escalation treatment with MPS, ultimately requiring unilateral hip replacement. Three patients developed acute psychosis (one case °III and two cases °IV according to common terminology criteria for adverse events). Whereas two cases resolved within days from MPS cessation, one patient required admission to psychiatry and long-term antipsychotic treatment.

In IA patients, hypocalcaemia (six patients, 38%) and hypotension (eight patients, 50%) were most common. Two cases of central venous catheter (CVC)-dislocation occurred in the IA group and two patients needed a femoral CVC. One case of °III CVC-associated septicaemia occurred (requiring vancomycin treatment.

### Assessment of peripheral blood immune cells during treatment

**Fig. 3:**
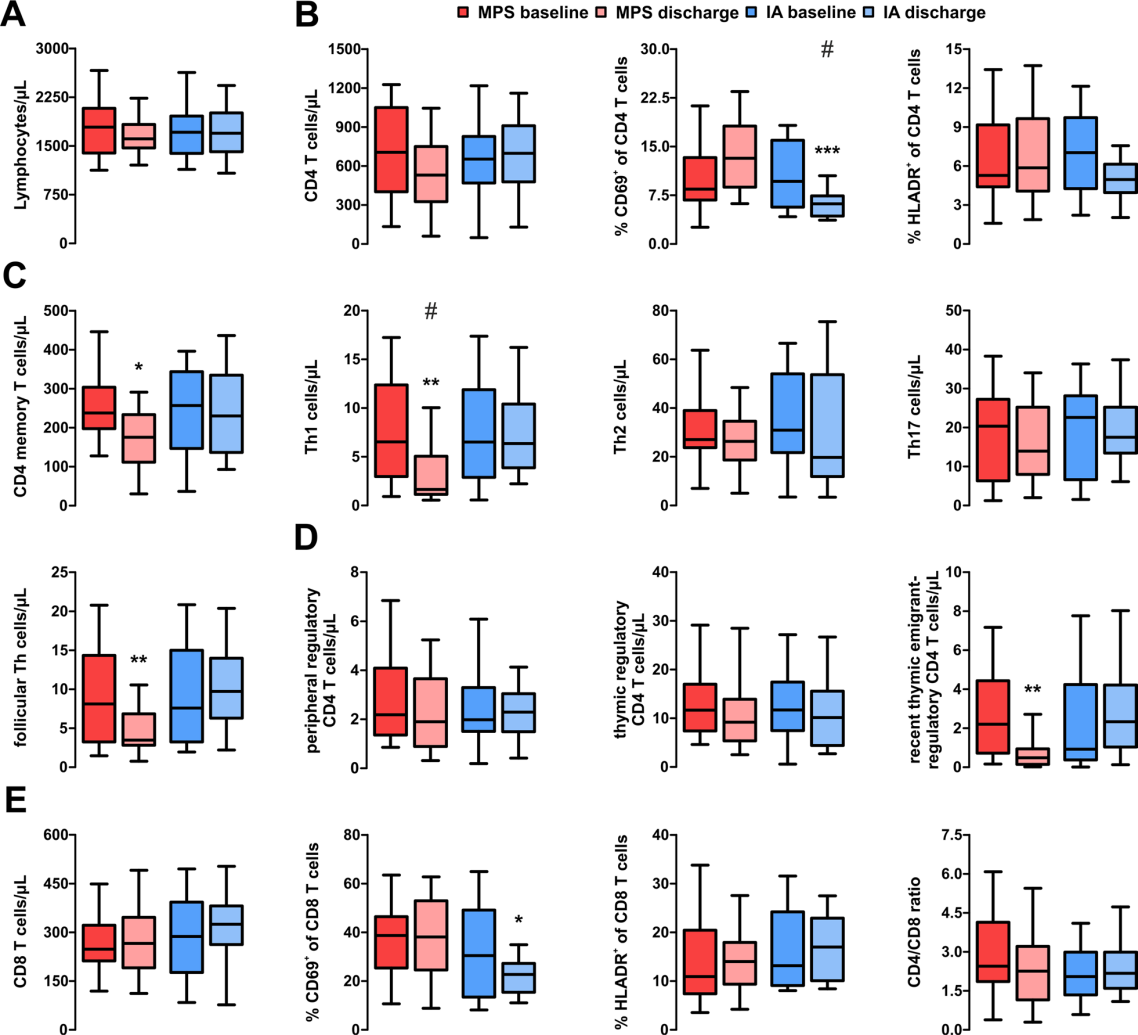
Flow cytometry analysis of T cells at baseline and discharge. **A** Blood lymphocyte count. **B** CD4 + T cells and activation markers (fraction of CD69 + and HLA-DR + cells). **C** Further CD4 + T cell subsets. **D** regulatory T cells subsets. **E** CD8 + T cells and activation markers. IA: 14 patients; MPS: 25 patients. Boxes indicate median ± IQR and whiskers indicate range. Significance evaluated using Kruskal–Wallis test including Dunn’s post-test. *: p < 0.05; **: p < 0.01; ***: p < 0.001. ; #p < 0.0031 (Bonferroni-corrected p-value)

Little is known about the immunologic effects of IA treatment in refractory RMS relapses and analyses are complicated by the assumption that different mediators are tackled by MPS (immune cells) and IA (soluble factors). For generation of deeper insights of the underlying mechanisms of either treatment, we first performed extensive peripheral blood mononuclear cell analysis at discharge and follow-up. 39 patients provided complete flow cytometry datasets and were further evaluated (MPS: 17 patients; MPS + IA: eight patients; IA: 14 patients). Total lymphocyte counts remained stable throughout (median: 1600 cells/µL at discharge; Fig. 3A).

Among CD4 + T cells, a relevant reduction of CD4 + memory T cells (176/µL vs. 238/µL at baseline; *p* = 0.025) as well Th1 cells (1.6/µL vs. 6.5/µL; *p* = 0.003) and follicular T helper cells (3.5/µL vs. 8.1/µL*; p* = 0.007) following MPS treatment was observed, whereas IA had no effect. Interestingly, MPS patients showed ongoing suppression of recent-thymic emigrant regulatory T cells (0.9/µL vs. 2.3/µL*; p* = 0.005), eventually due to the more recent steroid exposure (Fig. 3B–E).

Notably, IA was associated with a profound reduction in the rapid-responding activation marker CD69 on both CD4 + T cells (9.7% vs 6.2% at baseline; *p* < 0.001) and CD8 + T cells (23% vs 30%; *p* = 0.033), whereas HLA-DR expression remained stable.

**Fig. 4:**
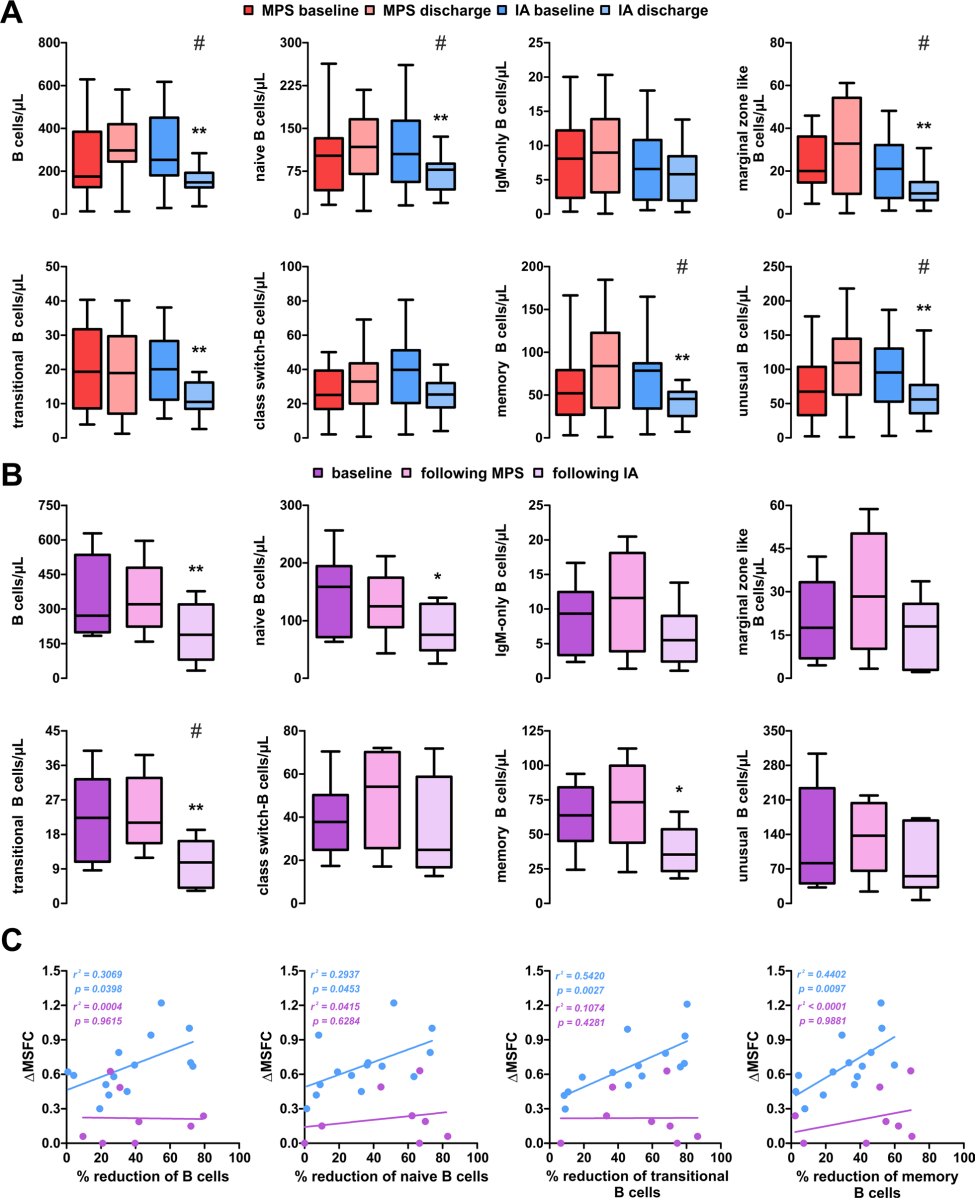
Flow cytometry analysis of the B cell compartment. **A** Development of B cell subsets. Significance evaluated using the Mann–Whitney test. IA: 14 patients; MPS: 26 patients. **B **Development of B cell subsets in patients undergoing IA following two courses of MPS [8]. Boxes indicate median ± IQR and whiskers indicate range. Significance evaluated using Friedmann’s test including post-test. *: p < 0.05; *: p < 0.01. **C** association of treatment-related reduction in B cells with an increase in clinical function, assessed via MSFC, in patients with IA (n = 14) and MPS + IA (*n* = 8) using linear regression. *MPS* methylprednisolone, *IA* immunoadsorption, *MSFC* multiple sclerosis functional composite

Surprisingly, IA treatment also resulted in profound changes among B cell subsets. Total B cell count decreased following IA treatment (148/µL vs 253/µL at baseline; *p* = 0.002). Among B cell subsets, naïve B cells (78/µL vs 105/µL*; p* = 0.004), marginal zone-like B cells (9/µL vs 21/µL*; p* = 0.002), transitional B cells (8/µL vs 11/µL*; p* = 0.009), memory B cells (46/µL vs 79/µL*; p* = 0.003) and unusual (CD21^−/low^CD38^−^IgD^+^IgM^+^) B cells (36/µL vs 53/µL*; p* = 0.003) were decreased in IA patients at discharge (Fig. 4A). Similar trends were observed in MPS + IA patients (Fig. 4B).

We performed linear regression comparing the MSFC score increase with relative reduction of the different B cell subsets. Our models revealed a significant correlation between the relative reduction of either B cell subset analysed and improvement in clinical function. Effects were most pronounced for transitional B cells (1/slope = 112.5; *r*^*2*^ = 0.542; *p* = 0.003) and memory B cells (1/slope = 149.9; *r*^*2*^ = 0.440; *p* = 0.010), followed by total B cells (1/slope = 174.3*; r*^*2*^ = 0.307*; p* = 0.040) and naïve B cells (1/slope = 183.1; *r*^*2*^ = 0.294*; p* = 0.045). Surprisingly, this correlation was not observable in most MPS + IA patients (Fig. 4C).

**Fig. 5:**
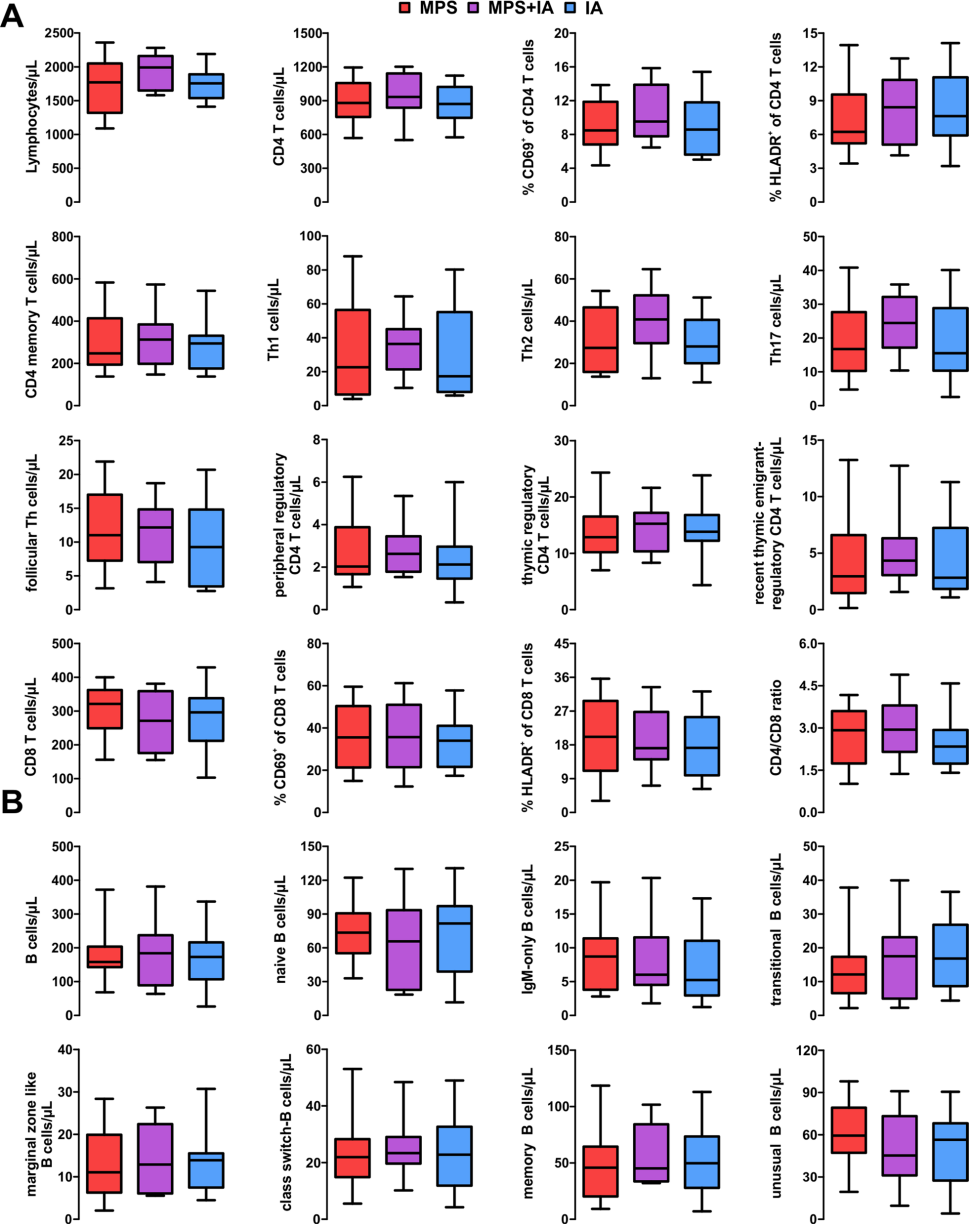
Flow cytometry analysis at follow-up. **A** Development of T cell subsets. Significance evaluated using the Mann–Whitney test. IA: 14 patients; MPS: 26 patients. **B** Development of B cell subsets. Boxes indicate median ± IQR and whiskers indicate range. Significance evaluated using Friedmann’s test including post-test. *: p < 0.05; *: p < 0.01. *MPS* methylprednisolone, *IA* immunoadsorption

Surprisingly, flow cytometry showed near-complete resolution of the effects observed at discharge, including restitution of T and B cells, among the different patient groups (Fig. 5).

#### Analysis of soluble factors

**Fig.6:**
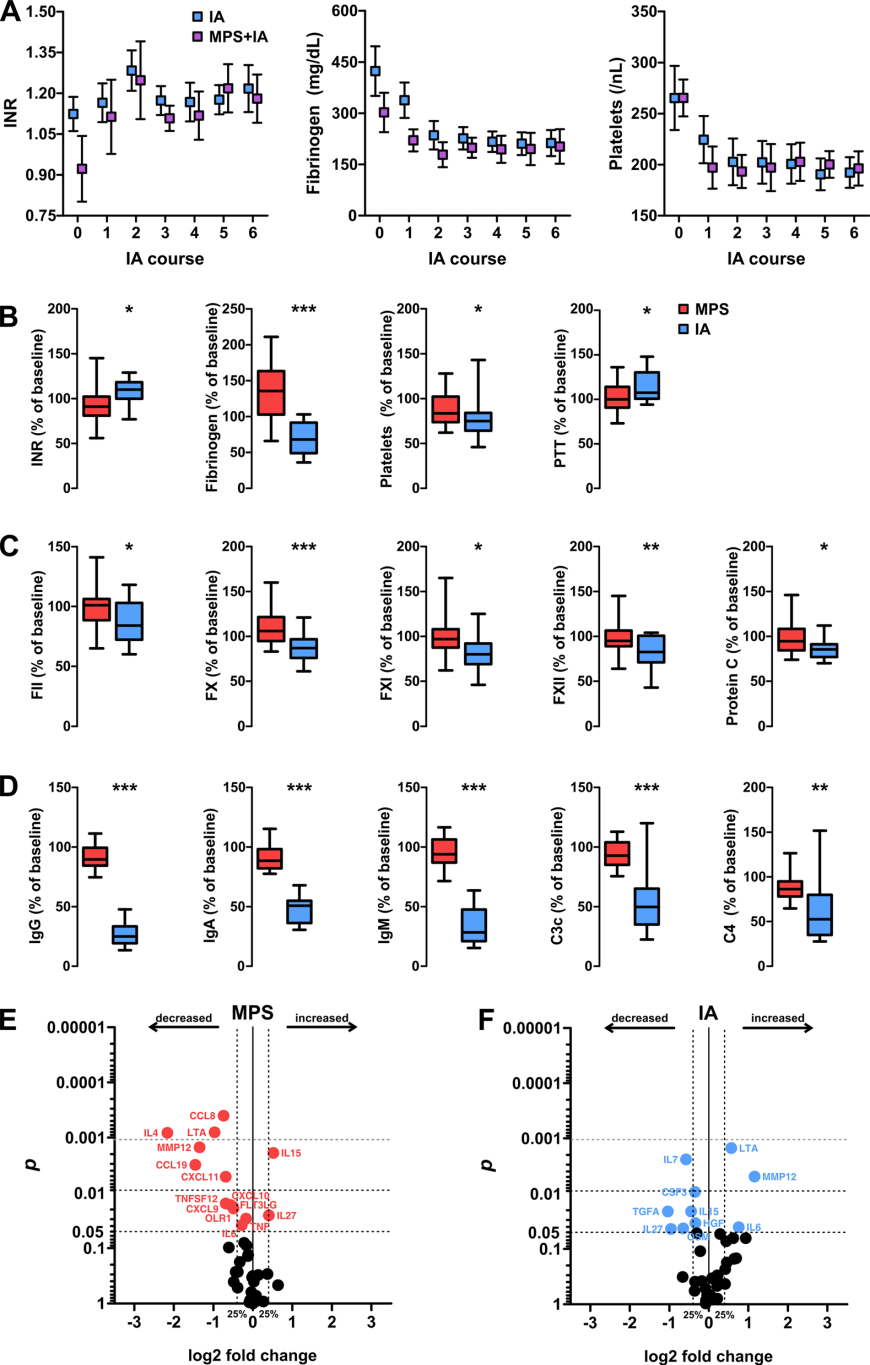
Serum soluble factor analysis including the OLINK™ target 48 cytokine panel. **A:** development of coagulation factors throughout IA treatment. Data are shown as mean ± SEM. **B** coagulation factors at discharge in MPS and IA patients (whiskers span from min to max). **C:** Determination of specific coagulation factors at discharge. **D** Analysis of immunoglobulin and complement levels at discharge. **E**, **F** OLINK multiplex cytokine analysis. Data are shown as volcano plots indicating the p-value of a Wilcoxon paired rank-sum test against the log2 fold change of cytokine levels at discharge compared to baseline (MPS: 26 patients; IA: 16 patients). Horizontal dashed lines indicate significance thresholds and vertical dashed lines indicate a fold-change exceeding ± 25% of baseline values. Tabular results are shown in Additional file 1: Table S2

Since coagulopathy was been previously described during apheresis treatment [7, 9], an extensive analysis of coagulation factors was performed. We neither observed thromboembolic events nor significant bleeding. Although international normalized ratio levels were elevated immediately following apheresis, accompanied by dropping fibrinogen and platelet levels, none of the parameters notably exceeded reference ranges (Fig. 6A).

All of these parameters stabilised around the fourth course of IA treatment in IA and MPS + IA patients. At discharge, international normalized ratio levels decreased to 91% of baseline in IA patients and in the MPS group increased by 10%; *p* = 0.010 for comparison of IA and MPS. Fibrinogen decreased following IA (68% of baseline) and increased following MPS (136% of baseline; *p* < 0.001 for comparison of IA & MPS). Platelet count decreased in both groups (IA: 75%; MPS: 84%*; p* = 0.042) during treatment (Fig. 6B). We investigated further coagulation factors and again found relative decreases in IA patients, with the strongest effect seen for factors X (IA: 106%; MPS: 87%; *p* < 0.001) and XII (IA: 95%; MPS: 83%; *p* = 0.003; Fig. 6C).

Finally, we assessed for decreases in immunoglobulin levels and observed the well-described pattern [9] associated with tryptophan-IA (IgG: 25% of baseline; IgA: 51% of baseline; IgM: 29% of baseline), with complement factors decreasing simultaneously (C3c: 50% of baseline; C4: 53% of baseline). Neither immunoglobulin nor complement factor levels were notably altered by MPS treatment (Fig. 6D).

To complement the extensive flow cytometry analysis of our cohort, we decided to run larger-scale serum chemo-and cytokine profiling, including 45 chemo- and cytokines at baseline and at discharge among the MPS and IA patients. Results are displayed as volcano-plots in Fig. 6E, F (for tabular results see Additional file 1: Table S2). Generally, both treatments visibly altered cytokine networks. It is unclear whether cytokine levels were affected by the modulation of immune cells or—in the context of IA—if they were simply removed from the circulation by non-specific binding to the tryptophan columns. However, we were able to identify three different subgroups of cytokines. Firstly, we found cytokines that were predominantly reduced following MPS treatment, but not following IA: chemokines CCL8, CXCL9, CXCL10, and CXCL11 were identified here as well as IL4. Secondly, we found cytokines that were reduced following IA but not following MPS. This group included IL7, oncostatin M (OSM) and transforming-growth factor A (TGFA). IL13 and granulocyte colony-stimulating factor (CSF3) were reduced following IA and MPS treatment, but to a far lesser extent for the combination. Finally, seven cytokines were differentially regulated following each respective treatment. IA increased the levels of lymphotoxin alpha (LTA), matrix metalloproteinase 1 (MMP1), Fms-related tyrosine kinase 3 ligand (FLT3LG), IL6, and CCL19, whereas MPS treatment led to a decrease in these cytokines at discharge. Conversely, IL15 and IL27 were both reduced by IA treatment but increased by MPS treatment. Among the further 29 evaluated proteins, we did not observe notable effects.

## Discussion

IA is often regarded as alternative to escalated MPS or therapeutic plasma exchange in patients with acute RMS, yet high-level evidence regarding its effectiveness and safety profile is lacking [6]. Our data demonstrate favourable outcomes in patients having received tryptophan-IA compared to escalated MPS and these results persisted at three-month follow-up, including clinical function scores, health-related QoL assessments and serum NfL levels.

The safety profile was in accordance with previous reports, with complications of CVC usage (dislocation, necessity of a femoral CVC) requiring the most clinical attention. Our study also highlighted safety concerns regarding escalated MPS, including not only hyperglycaemia, hypokalaemia and hypertension but also the development of severe psychosis and infection.

Furthermore, we documented profound changes in peripheral immune cell composition, most notably a decrease of B cells and reduction of T cell activation markers following apheresis treatment contrasting isolated changes in the T cell compartment following double-dose MPS. We also documented depletion of various soluble factors including immunoglobulins, coagulation factors and cytokines following immunoadsorption.

Apheresis treatment was first established for inflammatory demyelinating disorders of the central nervous system following a randomized, sham-controlled trial by Weinshenker and colleagues [19]. Several studies have been reported since, but were mostly retrospective and/or uncontrolled. IA was first studied for treatment of diseases thought to be primarily driven by autoantibodies, such as myasthenia gravis or autoimmune encephalitis [20, 21]. However, several studies reported alleviation of MS relapses following IA treatment [8, 13]. Notably, a blinded trial even demonstrated the superiority of IA over plasma exchange in MS [9]. Unfortunately, a larger randomized multicentre clinical trial comparing IA and escalated MPS was registered in 2018 yet results are pending (EudraCT: 2017-000635-13).

Several questions remain unanswered regarding immunoadsorption. First of all, it remains unclear by which mechanism IA alleviates relapse symptoms. Clearance of immunoglobulins has been deemed as most likely mechanism of apheresis treatment and indeed was supported by previous findings. The use of protein A columns was shown to be equally effective to the use of tryptophan columns and those columns are deemed highly specific for immunoglobulin G [6, 8]. Previous studies also identified the presence of immune complexes in the context of type II MS lesions essential for success of apheresis treatment in smaller studies further pointing towards antibody clearance as mechanism of action [22]. The other way around, experimental studies showed that passive transfer of antibodies separated from RMS patient’s plasma by protein A immunoadsorption was capable to aggravate rodent experimental autoimmune encephalitis [23].

However, apart from the study from Keegan and colleagues [22], apheresis treatment was successful in the vast majority of patients it was applied to and a substantial proportion of “non-responders” was absent although it should have been present considering the supposed association to a specific histotype. Furthermore, previous studies indicated that depletion of autoantibodies alone did not influence production of new antibodies indicating absence of relevant feedback-loops [24].

We found that IA not only influenced immunoglobulin levels but also exerted profound effects on blood lymphocytes. Specifically, a profound reduction of nearly all examined B cell subsets immediately following IA treatment was observed. Surface expression of the rapid-responding activation marker CD69 [25] on T cells also declined rapidly, whereas the slower-reacting HLA-DR expression remained unaffected indicating that the recent treatment was responsible here.

As the reduction of B cell subsets in the periphery correlated to clinical function outcomes, we assume that B cell-modulation is a central mechanism of IA. Notably, we found that administration of two courses of MPS prior to IA abrogated not only the observed correlation between B cell depletion and clinical outcomes but also hampered clinical recovery reflected by MSFC and EP scores, as well as being associated with a smaller reduction in NfL levels, at follow-up. The cause for this observation is unclear; however, it is known that high-dose MPS may modulate the blood–brain-barrier [26] and impair protein re-distribution in the blood, which might impair IA efficacy, as this therapy is thought to clear serum protein and lower the protein concentration including antibody levels [27]. Furthermore, patients in the MPS + IA group were latest to receive IA since relapse onset and thus, one could also assume that the “window of opportunity” during which modulation of the immune system can result in alleviation of neurologic deficits, had closed.

Previously, a threshold of six weeks from relapse onset was discussed as suitable for initial corticosteroid treatment [4, 28, 29]. yet no data regarding escalation treatment exist. Median time from relapse onset to apheresis treatment in our MPS + IA patient was still below this (median: 39 days), still, this subgroup of patients was refractory to two courses of treatment already und thus, persistent structural damage is already likely.

Interestingly, IA treatment shifted the cytokine repertoire compared to MPS treatment and reduced the cytokines necessary for B cell maturation as well as B cell-derived cytokines that are supposed to maintain neuroinflammation. For example, IA was associated with reduction of IL7 and IL15, which are both known to promote B cell-mediated recruitment of CD4 + and CD8 + T cells [30, 31]. Furthermore, IL27, which is thought to also contribute to B cell development [32], was reduced. Conversely, lymphotoxin alpha, which is considered as pro-inflammatory B-cell derived cytokine in MS [33], was reduced following MPS treatment but increased following IA treatment.

MPS-associated changes in the cytokine network comprised cytokines such as IL4, which is also pivotal to B cell maturation [34], and IL6, which is known as important effector cytokine secreted by several B cell subsets in MS patients [35]. However, these findings did not result in a substantial reduction of B cells.

Some of our findings regarding cytokines involved in B cell maturation and activation have been described in RMS already. Anti-CD20 therapy also induces changes in IL-7 and IL-15 levels [36] further implying that B-cell-mediated T cell activation is an important mechanism of B-cell-dependent inflammatory demyelination. In line with this, clinical data from anti-CD20 antibody trials showed that B cell depletion reduced the burden of contrast-enhancing MRI lesions early after treatment [37], supporting that B cell modulation can indeed resolve acute inflammation.

However, we observed that the specific effects of IA on B cell subsets disappeared within three months. Since re-emergence of peripheral B cell subsets is associated with disease reactivation in patients receiving B cell-depleting anti-CD20 treatment [38], protective effects of IA beyond month 3 appear unlikely. Conversely, effects of IA on the immune system including impaired response to vaccines as observed following B cell-depletion [39], are reversible.

Although not conventionally randomized, we aimed for reduction of potential bias by various mechanisms. First of all, treatment decision was made independently from study conduction using a standardized decision-making process led by neutral consultants. Second, we evaluated only patients with their first refractory MS relapse and moreover, more than half of patients experienced their first clinical demyelinating event ever further reducing a potential treatment-bias. In line with this, the majority of patients were treatment-naïve. Among patients already receiving disease-modifying treatment, substances were equally distributed. None of the patients received cell-depleting therapy. Although sample size of previously-treated patients remains too low for distinct subgroup analysis, those patients showed courses and laboratory findings similar to their naïve counterparts. Further evaluation of baseline epidemiological parameters showed no relevant differences among groups. We of course cannot rule out that indeed, a certain degree of restitution is a delayed effect of initial MPS treatment; however, this would not explain differences between IA and MPS escalation treatment.

In conclusion, IA proved to be a promising strategy for steroid-refractory RMS and thus should be considered early in treatment algorithms. Since the safety profile appeared advantageous to plasma exchange in previous reports and effectiveness appeared superior in outcomes such as MSFC, its use in routine clinical practice should be considered, especially in specialized centres. Furthermore, our findings indicate that modulation of B cells potentially represents a major mechanism of action of IA treatment.

## Supplementary Information

**Additional file 1:** **Table S1. **Antibodies used for flow cytometric analysis of PBMC. **Table S2.** Tabular results for OLINK™ target 48-cytokine screening. Significance testing was performed using Wilcoxon’s paired rank-sum test.

## Abbreviations

CVC: Central venous catheter
EDSS: Expanded disability status scale
IA: Immunoadsorption
IL: Interleukin
MPS: Methylprednisolone
MSFC: Multiple sclerosis functional composite
NfL: Neurofilament light chain
QoL: Quality of life
RMS: Multiple sclerosis

## References

[1] Berkovich RR (2016). Acute multiple sclerosis relapse. Continuum (Minneap Minn).

[2] V. DGfrNDe. S2k-leitlinie, diagnose und therapie der multiplen sklerose, neuromyelitis optica spektrum und MOG-IgG- assoziierte Erkrankungen. 2020. https://dgn.org/wp-content/uploads/2020/09/200902_MS-LL_Hauptteil_Konsultationsfassung_KKNMS_202008_final.pdf. Acessed 21 June 2022.

[3] Sellebjerg F, Barnes D, Filippini G, Midgard R, Montalban X, Rieckmann P (2005). EFNS guideline on treatment of multiple sclerosis relapses: report of an EFNS task force on treatment of multiple sclerosis relapses. Eur J Neurol.

[4] Stoppe M, Busch M, Krizek L, Then BF (2017). Outcome of MS relapses in the era of disease-modifying therapy. BMC Neurol.

[5] Oliveri RL, Valentino P, Russo C, Sibilia G, Aguglia U, Bono F (1998). Randomized trial comparing two different high doses of methylprednisolone in MS: a clinical and MRI study. Neurology.

[6] Rolfes L, Pfeuffer S, Ruck T, Melzer N, Pawlitzki M, Heming M (2019). Therapeutic apheresis in acute relapsing multiple sclerosis: current evidence and unmet needs-a systematic review. J Clin Med.

[7] Pfeuffer S, Rolfes L, Bormann E, Sauerland C, Ruck T, Schilling M (2019). Comparing plasma exchange to escalated methyl prednisolone in refractory multiple sclerosis relapses. J Clin Med.

[8] Koziolek MJ, Tampe D, Bahr M, Dihazi H, Jung K, Fitzner D (2012). Immunoadsorption therapy in patients with multiple sclerosis with steroid-refractory optical neuritis. J Neuroinflammation.

[9] Dorst J, Fangerau T, Taranu D, Eichele P, Dreyhaupt J, Michels S (2019). Safety and efficacy of immunoadsorption versus plasma exchange in steroid-refractory relapse of multiple sclerosis and clinically isolated syndrome: a randomised, parallel-group, controlled trial. EClinicalMedicine.

[10] Mauch E, Zwanzger J, Hettich R, Fassbender C, Klingel R, Heigl F (2011). Immunoadsorption for steroid-unresponsive multiple sclerosis-relapses: clinical data of 14 patients. Nervenarzt.

[11] Schimrigk S, Faiss J, Kohler W, Gunther A, Harms L, Kraft A (2016). Escalation therapy of steroid refractory multiple sclerosis relapse with tryptophan immunoadsorption—observational multicenter study with 147 patients. Eur Neurol.

[12] Trebst C, Bronzlik P, Kielstein JT, Schmidt BM, Stangel M (2012). Immunoadsorption therapy for steroid-unresponsive relapses in patients with multiple sclerosis. Blood Purif.

[13] Heigl F, Hettich R, Arendt R, Durner J, Koehler J, Mauch E (2013). Immunoadsorption in steroid-refractory multiple sclerosis: clinical experience in 60 patients. Atheroscler Suppl.

[14] Padmanabhan A, Connelly-Smith L, Aqui N, Balogun RA, Klingel R, Meyer E, et al. Guidelines on the use of therapeutic apheresis in clinical practice—evidence-based approach from the Writing Committee of the American Society for Apheresis: The Eighth Special Issue. J Clin Apher. 2019;34(3):171–354. 10.1002/jca.21705 31180581

[15] Schmidt J, Gold R, Schonrock L, Zettl UK, Hartung HP, Toyka KV (2000). T-cell apoptosis in situ in experimental autoimmune encephalomyelitis following methylprednisolone pulse therapy. Brain.

[16] Jung P, Beyerle A, Ziemann U (2008). Multimodal evoked potentials measure and predict disability progression in early relapsing-remitting multiple sclerosis. Mult Scler.

[17] Conway BL, Zeydan B, Uygunoglu U, Novotna M, Siva A, Pittock SJ (2018). Age is a critical determinant in recovery from multiple sclerosis relapses. Mult Scler.

[18] Thompson AJ, Banwell BL, Barkhof F, Carroll WM, Coetzee T, Comi G (2018). Diagnosis of multiple sclerosis: 2017 revisions of the McDonald criteria. Lancet Neurol.

[19] Weinshenker BG, O'Brien PC, Petterson TM, Noseworthy JH, Lucchinetti CF, Dodick DW (1999). A randomized trial of plasma exchange in acute central nervous system inflammatory demyelinating disease. Ann Neurol.

[20] Schneider-Gold C, Krenzer M, Klinker E, Mansouri-Thalegani B, Mullges W, Toyka KV (2016). Immunoadsorption versus plasma exchange versus combination for treatment of myasthenic deterioration. Ther Adv Neurol Disord.

[21] Heine J, Ly LT, Lieker I, Slowinski T, Finke C, Pruss H (2016). Immunoadsorption or plasma exchange in the treatment of autoimmune encephalitis: a pilot study. J Neurol.

[22] Keegan M, Konig F, McClelland R, Bruck W, Morales Y, Bitsch A (2005). Relation between humoral pathological changes in multiple sclerosis and response to therapeutic plasma exchange. Lancet.

[23] Pedotti R, Musio S, Scabeni S, Farina C, Poliani PL, Colombo E (2013). Exacerbation of experimental autoimmune encephalomyelitis by passive transfer of IgG antibodies from a multiple sclerosis patient responsive to immunoadsorption. J Neuroimmunol.

[24] Goldammer A, Derfler K, Herkner K, Bradwell AR, Horl WH, Haas M (2002). Influence of plasma immunoglobulin level on antibody synthesis. Blood.

[25] Reddy M, Eirikis E, Davis C, Davis HM, Prabhakar U (2004). Comparative analysis of lymphocyte activation marker expression and cytokine secretion profile in stimulated human peripheral blood mononuclear cell cultures: an in vitro model to monitor cellular immune function. J Immunol Methods.

[26] Gold R, Buttgereit F, Toyka KV (2001). Mechanism of action of glucocorticosteroid hormones: possible implications for therapy of neuroimmunological disorders. J Neuroimmunol.

[27] Dogan Onugoren M, Golombeck KS, Bien C, Abu-Tair M, Brand M, Bulla-Hellwig M (2016). Immunoadsorption therapy in autoimmune encephalitides. Neurol Neuroimmunol Neuroinflamm.

[28] Ontaneda D, Rae-Grant AD (2009). Management of acute exacerbations in multiple sclerosis. Ann Indian Acad Neurol.

[29] Ramo-Tello C, Blanco Y, Brieva L, Casanova B, Martinez-Caceres E, Ontaneda D (2021). Recommendations for the Diagnosis and Treatment of Multiple Sclerosis Relapses. J Pers Med..

[30] Li R, Rezk A, Healy LM, Muirhead G, Prat A, Gommerman JL (2015). Cytokine-defined B cell responses as therapeutic targets in multiple sclerosis. Front Immunol.

[31] Lee LF, Axtell R, Tu GH, Logronio K, Dilley J, Yu J (2011). IL-7 promotes T(H)1 development and serum IL-7 predicts clinical response to interferon-beta in multiple sclerosis. Sci Transl Med..

[32] Larousserie F, Charlot P, Bardel E, Froger J, Kastelein RA, Devergne O (2006). Differential effects of IL-27 on human B cell subsets. J Immunol.

[33] McWilliam O, Sellebjerg F, Marquart HV, von Essen MR (2018). B cells from patients with multiple sclerosis have a pathogenic phenotype and increased LTalpha and TGFbeta1 response. J Neuroimmunol.

[34] Granato A, Hayashi EA, Baptista BJ, Bellio M, Nobrega A (2014). IL-4 regulates Bim expression and promotes B cell maturation in synergy with BAFF conferring resistance to cell death at negative selection checkpoints. J Immunol.

[35] Barr TA, Shen P, Brown S, Lampropoulou V, Roch T, Lawrie S (2012). B cell depletion therapy ameliorates autoimmune disease through ablation of IL-6-producing B cells. J Exp Med.

[36] de Flon P, Soderstrom L, Laurell K, Dring A, Sundstrom P, Gunnarsson M (2018). Immunological profile in cerebrospinal fluid of patients with multiple sclerosis after treatment switch to rituximab and compared with healthy controls. PLoS ONE.

[37] Hauser SL, Bar-Or A, Comi G, Giovannoni G, Hartung HP, Hemmer B (2017). Ocrelizumab versus interferon beta-1a in relapsing multiple sclerosis. N Engl J Med.

[38] Ellrichmann G, Bolz J, Peschke M, Duscha A, Hellwig K, Lee DH (2019). Peripheral CD19(+) B-cell counts and infusion intervals as a surrogate for long-term B-cell depleting therapy in multiple sclerosis and neuromyelitis optica/neuromyelitis optica spectrum disorders. J Neurol.

[39] Bar-Or A, Calkwood JC, Chognot C, Evershed J, Fox EJ, Herman A (2020). Effect of ocrelizumab on vaccine responses in patients with multiple sclerosis: the VELOCE study. Neurology.

