# Supplementary material for: Immunoadsorption versus double-dose methylprednisolone in refractory multiple sclerosis relapses
Source: J Neuroinflammation. 2022 Sep 7;19:220. doi: 10.1186/s12974-022-02583-y (PMC9450381; doi:10.1186/s12974-022-02583-y)
Supplement: Supplementary file 1 — Additional file 1: Table S1. Antibodies used for flow cytometric analysis of PBMC. Table S2. Tabular results for OLINK™ target 48-cytokine screening. Significance testing was performed using Wilcoxon’s paired rank-sum test. [file 12974_2022_2583_MOESM1_ESM.docx]

| **Fluorochrome** | **Target antigen** | **Clone** | **Manufacturer** | **Lot** |
| --- | --- | --- | --- | --- |
| FITC | IgD | IA6-2 | Biolegend | B313580 |
| FITC | CD1c | AD5-8E7 | Miltenyi | 5200210910 |
| FITC | CD183/CXCR3 | G025H7 | Biolegend | B298571 |
| FITC | Helios | 22F6 | Biolegend | B224436 |
| PE | CD69 | TP1.55.3 | Beckman Coulter | 40 |
| PE | CD80 | L307.4 | BD Biosciences | 8005921 |
| PE | CD279/PD1 | eBioJ105 | eBiosciences | 2009785 |
| PE | FoxP3 | PCH101 | eBiosciences | 2189793 |
| ECD | CD24 | ALB9 | Beckman Coulter | 200044 |
| ECD | CD56 | N901 | Beckman Coulter | 200023 |
| ECD | HLA-DR | Immu-357 | Beckman Coulter | 200018 |
| PerCP/Cy5.5 | CD31 | WM59 | Biolegend | B272399 |
| PerCP/Cy5.5 | CD38 | HIT2 | Biolegend | B301400 |
| PerCP/Cy5.5 | CD194/CCR4 | TG6/CCR4 | Biolegend | B247086 |
| PerCP/Cy5.5 | Vα24-Jα18 | 6B11 | Biolegend | B282761 |
| PE/Cy7 | CD27 | 1A4CD27 | Beckman Coulter | 200028 |
| PE/Cy7 | CD56 | N901 | Beckman Coulter | 200075 |
| PE/Cy7 | CD196/CCR6 | 11A9 | BD Pharmingen | 9346308 |
| PE/Cy7 | TIGIT | MBSA43 | eBiosciences | 4330435 |
| APC | CD4 | 13B8.2 | Beckman Coulter | 200072 |
| APC | CD23 | EBV CS-5 | Biolegend | B173800 |
| APC | CD226/DNAM-1 | 11A8 | Biolegend | B223427 |
| APC | Streptavidin |  | Biolegend | B266052 |
| APC-A700 | CD8 | B9.11 | Beckman Coulter | 200024 |
| APC-A700 | CD19 | J3-119 | Beckman Coulter | 200035 |
| APC-A700 | CD28 | CD28.2 | Biolegend | B262655 |
| APC-A700 | CD127 | R34.34 | Beckman Coulter | 200052 |
| APC-A750 | CD3 | UCHT1 | Beckman Coulter | 200074 |
| APC-A750 | CD16 | 3G8 | Beckman Coulter | 200027 |
| APC-Fire750 | CD20 | 2H7 | Biolegend | B272721 |
| PacB | CD8 | B9.11 | Beckman Coulter | 200027 |
| VioBlue | CD21 | HB5 | Miltenyi | 5160704442 |
| BV421 | CD39 | A1 | Biolegend | B308983 |
| BV421 | CD278/ICOS | C398.4A | Biolegend | B293039 |
| BV510 | CD8 | SK1 | Biolegend | B315894 |
| BV510 | CD14 | M5E2 | Biolegend | B306943 |
| BV510 | CD146 | P1H12 | BD Biosciences | 174899 |
| BV510 | IgM | MHM-88 | Biolegend | B250599 |
| BV605 | CD45RO | UCHL1 | Biolegend | B314850 |
| BV650 | CD3 | UCHT1 | Biolegend | B301735 |
| BV650 | CD25/IL-2Rα | BC96 | Biolegend | B291221 |
| BV650 | CD27 | O323 | Biolegend | B273921 |
| BV785 | CD4 | Okt 04 | Biolegend | B313176 |
| Biotin | CD185/CCR5 | J252D4 | Biolegend | B236597 |

**Tab.S1:** Antibodies used for flow cytometric analysis of PBMC. FITC: fluorescein isothiocyanate; PE: phycoerythrin; ECD: electron-coupled dye; APC: allophycocyanin; PacB: pacific blue; BV: brilliant violet.

|  | **IA (n=16)** | | **MPS (n=26)** | |
| --- | --- | --- | --- | --- |
|  | ***log2 fold-change*** | ***p*** | ***log2 fold change*** | ***p*** |
| **CCL2** | *0,42* | *0,233* | *-0,33* | *0,179* |
| **CCL3** | *0,21* | *0,470* | *-0,38* | *0,270* |
| **CCL4** | *0,21* | *0,791* | *-0,07* | *0,872* |
| **CCL7** | *0,41* | *0,432* | *-0,48* | *0,409* |
| **CCL8** | *-0,07* | *0,850* | *-0,74* | *0,001* |
| **CCL11** | *-0,22* | *0,110* | *0,08* | *0,727* |
| **CCL13** | *-0,31* | *0,052* | *-0,16* | *0,090* |
| **CCL19** | *0,62* | *0,064* | *-1,46* | *0,003* |
| **CXCL8** | *0,69* | *0,148* | *-0,02* | *0,339* |
| **CXCL9** | *0,20* | *0,322* | *-0,50* | *0,018* |
| **CXCL10** | *-0,05* | *0,677* | *-0,57* | *0,017* |
| **CXCL11** | *-0,08* | *0,970* | *-0,69* | *0,005* |
| **CXCL12** | *0,20* | *0,791* | *-0,13* | *0,139* |
| **CSF1** | *0,03* | *0,677* | *0,00* | *0,320* |
| **CSF2** | *-0,35* | *0,391* | *0,37* | *0,294* |
| **CSF3** | *-0,35* | *0,009* | *-0,39* | *0,518* |
| **IL1b** | *-0,05* | *0,761* | *-0,86* | *0,324* |
| **IL2** | *-0,84* | *0,839* | *-0,67* | *0,732* |
| **IL4** | *0,21* | *0,110* | *-2,03* | *0,001* |
| **IL6** | *0,76* | *0,041* | *-0,61* | *0,098* |
| **IL7** | *-0,58* | *0,002* | *0,14* | *0,307* |
| **IL10** | *-0,66* | *0,322* | *0,26* | *0,926* |
| **IL13** | *-1,33* | *0,021* | *-0,44* | *0,018* |
| **IL15** | *-0,45* | *0,021* | *0,52* | *0,002* |
| **IL17A** | *0,93* | *0,064* | *0,08* | *0,896* |
| **IL17C** | *0,08* | *0,339* | *0,00* | *1,000* |
| **IL17F** | *-0,65* | *0,153* | *0,18* | *0,666* |
| **IL18** | *-0,18* | *0,380* | *0,03* | *0,404* |
| **IL27** | *-0,96* | *0,044* | *0,41* | *0,026* |
| **IL33** | *0,42* | *0,952* | *-0,04* | *0,420* |
| **IFNG** | *-0,36* | *0,572* | *0,64* | *0,466* |
| **FLT3LG** | *0,64* | *0,151* | *-0,69* | *0,016* |
| **EGF** | *0,13* | *0,742* | *0,19* | *0,776* |
| **HGF** | *-0,34* | *0,034* | *-0,04* | *0,628* |
| **LTA** | *0,57* | *0,002* | *-0,97* | *0,001* |
| **MMP1** | *1,15* | *0,005* | *-1,35* | *0,002* |
| **MMP12** | *0,29* | *0,054* | *0,06* | *0,922* |
| **OLR1** | *0,45* | *0,176* | *-0,49* | *0,019* |
| **OSM** | *-0,65* | *0,043* | *-0,09* | *0,957* |
| **TGFA** | *-1,04* | *0,021* | *0,02* | *0,936* |
| **TNF** | *0,22* | *0,301* | *-0,28* | *0,038* |
| **TNFSF10** | *0,44* | *0,073* | *-0,12* | *0,132* |
| **TNFSF12** | *-0,08* | *0,519* | *-0,17* | *0,029* |
| **TSLP** | *-0,25* | *0,193* | *0,02* | *0,289* |
| **VEGFA** | *-0,01* | *0,791* | *-0,22* | *0,080* |

**Tab.S2:** Tabular results for OLINK™ target 48-cytokine screening. Significance testing was performed using Wilcoxon’s paired rank-sum test.
